# Acute expression of human APOBEC3B in mice results in RNA editing and lethality

**DOI:** 10.1186/s13059-023-03115-4

**Published:** 2023-11-24

**Authors:** Alicia Alonso de la Vega, Nuri Alpay Temiz, Rafail Tasakis, Kalman Somogyi, Lorena Salgueiro, Eleni Zimmer, Maria Ramos, Alberto Diaz-Jimenez, Sara Chocarro, Mirian Fernández-Vaquero, Bojana Stefanovska, Eli Reuveni, Uri Ben-David, Albrecht Stenzinger, Tanja Poth, Mathias Heikenwälder, Nina Papavasiliou, Reuben S. Harris, Rocio Sotillo

**Affiliations:** 1 Division of Molecular Thoracic Oncology, German Cancer Research Center (DKFZ), Im Neuenheimer Feld 280, 69120 Heidelberg, Germany; 2 Ruprecht Karl University of Heidelberg, 69120 Heidelberg, Germany; 3 Health Informatics Institute, University of Minnesota, Minneapolis, 55455 USA; 4 Division of Immune Diversity, German Cancer Research Center (DKFZ), Im Neuenheimer Feld 280, 69120 Heidelberg, Germany; 5 Division of Chronic Inflammation and Cancer, German Cancer Research Center (DKFZ), Heidelberg, Germany; 6 Department of Biochemistry and Structural Biology, University of Texas Health San Antonio, San Antonio, TX 78229 USA; 7 Howard Hughes Medical Institute, University of Texas Health San Antonio, San Antonio, TX 78229 USA; 8 Department of Human Molecular Genetics and Biochemistry, Faculty of Medicine, Tel Aviv University, Tel Aviv, Israel; 9 Institute of Pathology, University Hospital Heidelberg, Heidelberg, Germany; 10 Translational Lung Research Center Heidelberg (TRLC), German Center for Lung Research (DZL), Heidelberg, Germany

**Keywords:** APOBEC3B, RNA editing, Mutations, Mouse models, DNA damage

## Abstract

**Background:**

RNA editing has been described as promoting genetic heterogeneity, leading to the development of multiple disorders, including cancer. The cytosine deaminase APOBEC3B is implicated in tumor evolution through DNA mutation, but whether it also functions as an RNA editing enzyme has not been studied.

**Results:**

Here, we engineer a novel doxycycline-inducible mouse model of human APOBEC3B-overexpression to understand the impact of this enzyme in tissue homeostasis and address a potential role in C-to-U RNA editing. Elevated and sustained levels of APOBEC3B lead to rapid alteration of cellular fitness, major organ dysfunction, and ultimately lethality in mice. Importantly, RNA-sequencing of mouse tissues expressing high levels of APOBEC3B identifies frequent UCC-to-UUC RNA editing events that are not evident in the corresponding genomic DNA.

**Conclusions:**

This work identifies, for the first time, a new deaminase-dependent function for APOBEC3B in RNA editing and presents a preclinical tool to help understand the emerging role of APOBEC3B as a driver of carcinogenesis.

**Supplementary Information:**

The online version contains supplementary material available at 10.1186/s13059-023-03115-4.

## Background

RNA editing is emerging at the forefront of epitranscriptomics having a fundamental role in multiple human diseases, including cancer [1–4]. This mechanism generates changes at the RNA level regulating genetic plasticity and resulting in protein diversity. RNA editing is a co- or post-transcriptional modification that most frequently involves the conversion of cytosine to uracil (C-to-U) or adenosine to inosine (A-to-I) by APOBEC or ADAR family of deaminases, respectively [5]. Although A-to-I editing has been reported extensively in thousands of positions in different species, C-to-U editing has been investigated to lesser extent. The first C-to-U editing event was described in apolipoprotein B (*ApoB*) mRNA which results in a stop codon (UAA) and the generation of the truncated protein ApoB48 [6]. *ApoB* mRNA editing is associated with the metabolism of lipoproteins and impaired editing increases the risk of cardiovascular disease [7, 8]. To date, only a few studies have directly connected C-to-U conversion to biological processes including cancer [9–12]. Even though most edits occur in non-coding regions and could have no impact, these modifications may trigger novel splice sites, alter microRNAs activity, and/or mRNA stability. Interestingly, it has been shown that the impact of RNA editing on proteome diversity is comparable to, or even greater than, that of somatic mutations [13, 14].

RNA C-to-U editing activity appears to be restricted to a few APOBEC members. Apobec1, the first member identified as an RNA editing enzyme is responsible for the editing of *ApoB* mRNA [15]. Overexpression of Apobec1 in mice and rabbits leads to hepatocellular carcinomas showing hyper-editing of multiple cytosines at different sites other than the canonical one on the *ApoB* mRNA [16], suggesting that high levels of APOBEC1 result in the loss of editing fidelity. In addition, under hypoxic conditions and interferon stimulation, the upregulation of APOBEC3A (A3A) has been associated with increased RNA editing in macrophages [17]. Human tumors expressing high levels of A3A also exhibit significant mRNA editing [18]. Lastly, APOBEC3G (A3G) has shown editing activity in HEK293T cells and lymphocytes [19]. These deaminases are promiscuous when selecting their substrate as they have an intrinsic ability to deaminate single-stranded DNA cytosines [12, 17, 20–23]. In comparison, other family members such as AID and APOBEC3B (A3B) have only been described to function at the DNA level [24–26]. Although there is evidence that AID and A3B are capable of binding to single-stranded RNA, this interaction appears to inhibit (not promote) editing activity [27–29].

Many different cancer genomes also manifest high levels of single base substitution (SBS) mutations attributable to APOBEC-catalyzed DNA editing [25, 26, 30–33]. These mutations are biased toward TCA and TCT motifs and are mostly comprised of C-to-T transitions and C-to-G transversions (SBS2 and SBS13, respectively, in COSMIC) [34]. The two APOBEC3 family members responsible for DNA mutations are A3A and A3B [30, 35]. A3A and A3B expression leads to the accumulation of mutations as well as DNA damage, which may compromise cellular integrity [25, 33, 36–39]. Indeed, clonal DNA sequencing revealed that A3A and A3B may be expressed in episodic bursts suggesting that continuous expression of these deaminases may be toxic for cancer cells [40]. In line with these results, the presence of APOBEC mutational signatures in human tumors does not always correlate with expression levels of A3A/B [18, 40]. Unlike DNA mutations, RNA editing is a dynamic process which does not leave a permanent footprint and RNA edits disappear right after the responsible enzymes are no longer expressed or after the turnover of the edited transcripts. Indeed, a recent study indicated that A3A activity can be detected by monitoring RNA editing hotspots [18]. Although several studies have addressed the relevance of RNA editing in cancer and other diseases [3, 4] the complexity of capturing the labile editing scenario could have masked editing activity by other APOBEC members. Therefore, whether A3B represents an epigenetic threat to RNA and cellular integrity has yet to be investigated.

Here, we report the development of a novel doxycycline-inducible mouse model expressing the human A3B. Upon doxycycline administration, high and sustained A3B levels are achieved in different tissues which trigger rapid lethality. Whole exome and transcriptome analysis of *A3B*-expressing tissues identified hundreds of A3B-induced RNA editing events with 5’-UCC as a preferred sequence context with the first C edited. Some of the A3B-edited positions appear to be hotspots that occur in the same RNA substrate in several distinct tissues of different mice. Importantly, continuous expression of catalytically active A3B is required for detecting RNA editing, since complete silencing of the *A3B* transgene as well as overexpression of a deaminase mutant *A3B* results in the lack of editing. Together our results identify a new function for *A3B* in RNA editing, which expands our understanding of the broader APOBEC family of editing enzymes and provides new opportunities to investigate the consequences of *A3B* expression in cancer and other diseases.

## Results

### Generation of an inducible mouse model for human APOBEC3B

**Fig. 1:**
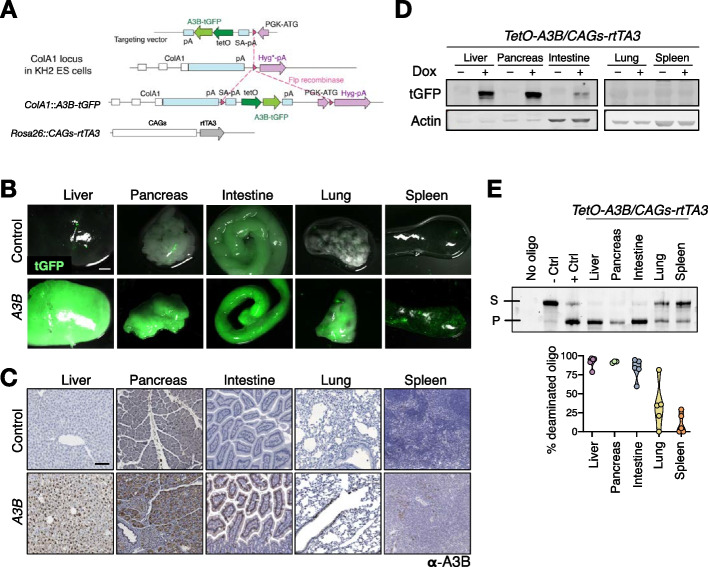
Generation of A3B-inducible mice. **A** Schematic representation of the strategy used for the generation of human A3B transgenic mice. Under a TRE promoter, the human *A3B* cDNA fused to *tGFP* was inserted after homologous recombination into the *ColA1* locus of KH2 ES cells. **B** Macroscopic images demonstrate tGFP fluorescence in tissues from *A3B* mice fed with doxycycline for 10 days (scale bar: 3 mm). **C** Immunohistochemistry of A3B in the indicated tissues from *A3B* mice fed with doxycycline for 10 days (scale bar: 100 μm). **D** Western blot analysis showing A3B levels in the indicated tissues from *A3B* mice with and without doxycycline treatment for 10 days. Anti-actin blots are shown as loading controls. **E** Representative DNA cytosine deaminase activity from whole cell extracts in the indicated tissues (S, Substrate; P, Product) and the corresponding quantification showing the % of the deaminated oligo (*n* = 5 mice per tissue)

To investigate the consequences of A3B activity in vivo, human *APOBEC3B* fused to turboGFP (hereafter *A3B*) was introduced into the *ColA1* locus of KH2 mouse embryonic stem cells [41] (Fig. 1A). In this system, *A3B* expression is placed under the control of a tetracycline-inducible operator (*tetO*) sequence. Mice containing *A3B* were crossed with a constitutive *CAGs-rtTA3* transgene (Additional file 1: Fig. S1A) that results in robust expression of the rtTA transactivator in adult tissues [42]. Adult 4-week-old *TetO-A3B-tGFP/CAGs-rtTA3* (*A3B*) mice were fed ab libitum with doxycycline (dox)-containing diet to systematically overexpress the *A3B* transgene. Ten days after daily doxycycline exposure human A3B was expressed most intensely in the liver and in the pancreas, with the enzyme localized predominantly to the nuclear compartment (Fig. 1B-D and Additional file 1: Fig. S1B-C). In comparison, intermediate levels of A3B protein were found in the intestine, and lower levels were detected in lung and spleen (Fig. 1B-D). We then sought to demonstrate if the *A3B* transgene retains its deaminase activity. Single-stranded DNA C-to-U activity assay with soluble protein extracts from *A3B*-expressing tissues demonstrated that A3B is functionally active (Fig. 1E) and the percentage of deaminated oligo is proportional to the amount of protein (Fig. S1D). To compare A3B expression levels in mice to those in humans, mRNA expression from livers, pancreas and lungs from *A3B* mice was normalized to the housekeeping gene encoding TATA-binding protein (TBP). Lung tissues showed A3B expression within the range observed across human cancers, while A3B expression in liver and pancreas was comparable to human tumors exhibiting the highest A3B levels which have been associated with poor survival in patients [43–45] (Additional file 1: Fig. S1E).

### Acute APOBEC3B induction in vivo is lethal

**Fig. 2:**
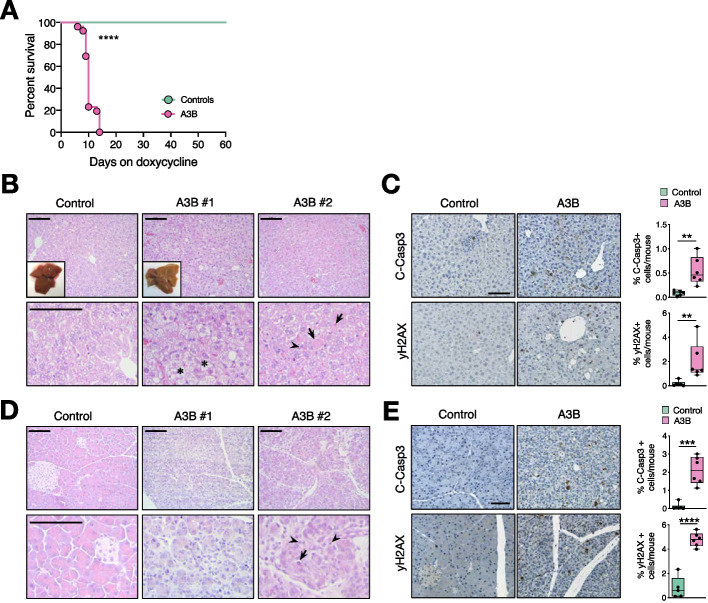
A3B overexpression causes early lethality. **A** Survival of *TetO-A3B/CAGs-rtTA3* mice after doxycycline administration (controls *n* = 9; *A3B n* = 26; *P* < 0.0001 by Log-rank (Mantel-Cox) test).** B** H&E-stained sections of livers from control and *A3B* mice (insets: macroscopic images). Asterisk point microvesicular steatosis, arrowhead point lymphocytes and arrows to apoptotic cells.** C** Immunohistochemistry against C-Caspase3 and γH2AX in liver sections from control and *A3B* mice and corresponding quantification (*n* = 6). **D** H&E-stained sections of the pancreas from control and *A3B* mice. Arrowheads point to lymphoplasmacellular infiltration and arrows to apoptotic cells. **E** Immunohistochemistry against C-Caspase3 and γH2AX in paraffin sections from control and *A3B* pancreas and the corresponding quantification (*n* = 6). Data in panels C and E were analyzed by unpaired t-test **p < 0.01, ***p < 0.001, ****p < 0.0001. Data is represented as mean ± SD shown by dots, where each dot represents a mouse, and error bars, respectively. Scale bars: 100 μm. Scale bars upper panels B and D: 10 μm

To examine the consequences of expressing A3B in vivo, dox-containing food was given to adult-4-week old *TetO-A3B/CAGs-rtTA3* mice. Unexpectedly, *A3B* mice showed a rapid health deterioration, and all animals died within 6 to 14 days after dox administration, while control animals were healthy beyond 365 days (Fig. 2A). Prior to death, A3B-expressing mice were largely unresponsive and immobile with a clear ‘‘trembling’’ phenotype. Macroscopically, *A3B* livers appeared indicative of fatty acid accumulation and close examination revealed a moderate to severe microvesicular steatosis (Fig. 2B), with few scattered lymphocytes and numerous apoptotic hepatocytes verified by cleaved caspase staining (Fig. 2C). Analysis of paraffin sections revealed also an increase in DNA damage (γH2AX staining) in *A3B* livers compared to controls whereas no differences in proliferation were observed (Additional file 1: Fig. S2A). Differential expression analysis of RNA-sequencing (RNA-seq) data comparing control and *A3B* expressing livers indicated a metabolic disturbance, due to the downregulation in the cholesterol metabolism (Additional file 1: Fig. S2B). Increased serum levels of liver enzymes, such as alanine transaminase (ALT) and aspartate transaminase (AST), also suggested liver damage (Additional file 1: Fig. S2C).

Parallel analyses of pancreatic sections, which also presented high levels of A3B, revealed a marked destruction of exocrine acinar cells with loss of zymogen granules, cellular shrinking, karyomegaly and a concomitant mild to moderate lymphocytic inflammation (Fig. 2D). Cleaved caspase staining clearly demonstrated an increase in apoptosis compared to control mice as well as an increase in γH2AX (Fig. 2E). These findings were accompanied with upregulation of inflammatory response pathways identified by differential expression analysis in the *A3B* pancreas compared to control (Additional file 1: Fig. S2D). Similar to liver tissues, no differences in proliferation (Additional file 1: Fig. S2E) or in lipase and amylase enzymes (Additional file 1: Fig. S2C) were found. Altogether, these results suggest that high levels of A3B lead to cell dysfunction, disruption of tissue homeostasis, systemic organ failure, and rapid animal death.

### APOBEC3B is an RNA editing enzyme

A3B has been implicated in the generation of DNA damage, mutagenesis, larger-scale chromosomal instability and phenotypic heterogeneity in cancer, mainly through its single-stranded DNA deamination activity [25, 35, 46]. The related A3A enzyme, which has high sequence similarity with the catalytic domain of A3B (92% identity), has been shown to be capable of editing RNA cytosines in primary human cells (macrophages) and human tumors [17, 18] further implicating this enzyme in tumor mutation and evolution [47–49]. We, therefore, explored whether A3B may be similarly capable of functioning as an RNA editing enzyme in vivo.

**Fig. 3:**
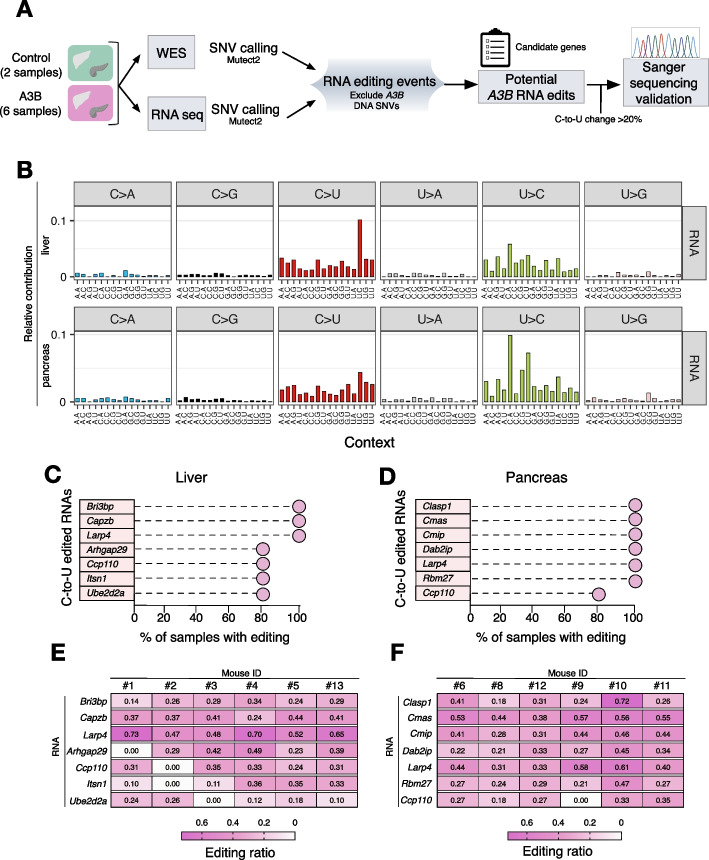
Local preference of APOBEC3B-driven RNA editing. **A** Schematic of the pipeline used to call RNA-editing sites in the A3B livers and pancreas. We used Mutect2 to identify DNA mutations and RNA edits. We pooled the WES and RNA-seq of the two control animals (no dox), and used them as a reference to identify variants of the A3B expressing tissues. This step was performed separately for DNA and RNA sequences, leading to the identification of DNA variants or RNA variants that were detected in at least one A3B expressing tissues but not in any of the other two control tissues. After that, we compared the RNA variants with the DNA variants from each sample to identify the RNA edits that are not DNA SNPs. **B** Trinucleotide mutation profiles for all base substitutions in the RNA from liver and pancreatic tissues of *A3B* mice (*n* = 6 in each group). Relative contribution refers to the contribution that each single base substitution has to the overall base substitution spectrum in *A3B* mice. **C-D** Lollipop plots indicating the percentage of mice showing C-to-U editing after experimental validation by RT-PCR in selected targets (*n* = 6 tissues in each group). **E–F** Heatmap plot showing the editing ratios of each sanger sequence validated position in each individual sample (liver shown in E and pancreas in F)

To test A3B RNA editing activity, RNA-seq and whole exome sequencing (WES) were performed in liver and pancreatic tissues (high A3B levels) from *A3B* mice after dox administration (10–14 days) as well as from similarly aged control littermates. RNA editing events were considered significant if they occurred in > 5% of RNA seq reads and were absent in whole exome DNA sequencing (WES) reads (Fig. 3A). Interestingly, high levels of C-to-U RNA editing were present in A3B-expressing tissues compared to controls. A-to-G (equivalent to T-to-C) RNA editing events were found in all RNA-seq specimens, most likely due to endogenous ADAR activity (Fig. 3B and Additional file 1: S3A,B). In comparison, DNA mutations were not as frequent and not biased to APOBEC signature motifs, which was not surprising because the rapid death of the mice did not allow for the generation of clonal mutations to be detected by bulk DNA sequencing (Additional file 1: Fig. S4A).

From the total RNA edits, we selected potential candidate genes (C-to-U > 20%) which were further validated to corroborate the reliability of our analysis (Fig. 3A and Additional file 2: Table S1). We identified 7 positions that showed C-to-U changes in the liver and 7 positions in the pancreas for experimental validation of site-specific editing by Sanger sequencing of purified RT-PCR RNA and DNA products (Additional file 1: Fig. S4B,C). In addition, to further explore whether the RNA editing events found in the *A3B* liver samples were random or recurrent events due to A3B overexpression, we sequenced these 7 edited positions in 6 additional *A3B*-expressing mice. We found that 3 of these positions were edited in 100% of the *A3B* mice, and 4 positions were edited in 5 out of 6 mice (83%) (Fig. 3C and Additional file 3: Table S2). In the pancreas, similar results were found, with 6 of the validated positions edited in 100% of the *A3B* mice, and 1 position edited in 5 out of 6 mice (83%) (Fig. 3D). Not surprisingly, editing was identified in mice that by RNA seq initially lacked a C-to-U change in a specific position, indicating that our analysis was stringent (VAF = 0.2) and that the editing landscape is indeed broader. Moreover, the editing ratios varied from 0 to 0.73 in the liver and from 0 to 0.72 in the pancreas (Fig. 3E,F). Altogether these results suggest that certain sites may be hotspots for A3B editing.

To explore whether moderate levels of A3B found in other tissues such as lung (Additional file 1: Fig. S4D) also induced RNA editing, we performed RNA-seq of 3 controls and 3 *A3B* lung tissues. After data processing and analysis, we generated a list of 15 potential candidate genes and experimental validation by Sanger sequencing of candidate C-to-U changes identified them as DNA SNPs (Additional file 1: Fig. S4E and Additional file 3: Table S2). In addition, we validated the recurrent candidate genes that were found to be edited in the liver, in lung samples from 3 mice (Additional file 3: Table S2). However, no editing was detected in any of these 7 positions, suggesting that moderate levels of A3B are not capable of inducing RNA editing at least at detectable levels by Sanger sequencing.

### APOBEC3B-driven RNA editing occurs at a specific hotspot

**Fig. 4:**
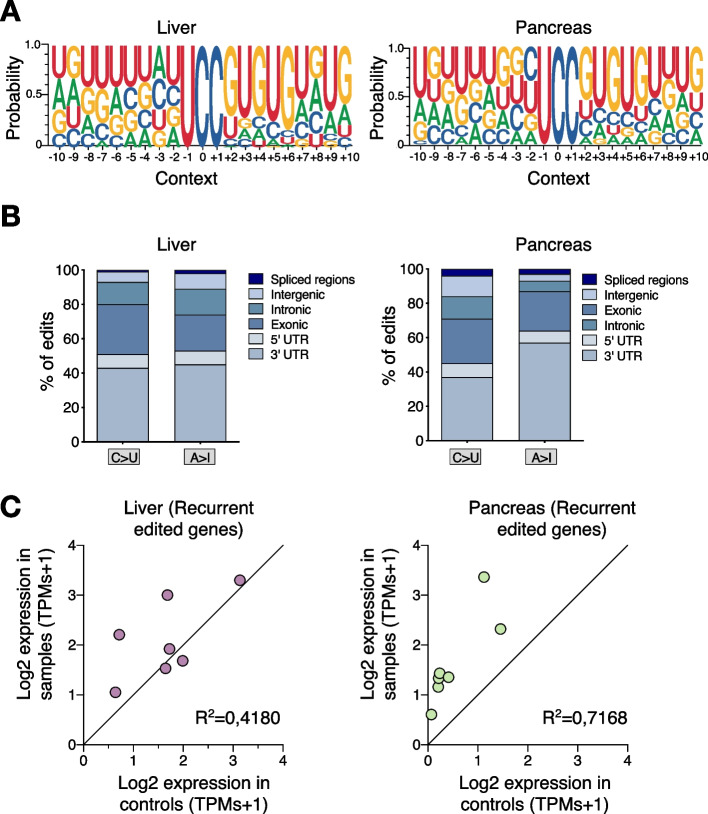
APOBEC3B-driven RNA editing occurs at a specific hotspot and mainly at 3’ UTRs. **A** Web logo representations of the broader sequence preferences surrounding the C-to-U editing events in 5’-UCC motifs in liver (left) and pancreas (right). **B** Distributions of the editing sites by region of the RNA editing events in liver (left) and pancreas (right). **C** Correlation of the expression levels in the recurrent edited transcripts in liver (left) and pancreas (right) samples obtained from RNA seq data (Log2 of transcripts per million + 1 (TPMs); each dot represents the average expression of each transcript (*n* = 2 controls and 6 *A3B* in each liver and pancreas samples)

The majority of the A3B-induced RNA changes were detected in a UCC context and all the edited genes that were validated in liver and pancreatic samples were identified in at least two animals. Therefore, we next explored whether A3B has any nucleotide preference surrounding the edited sequence. Analysis of the flanking nucleotides identified a broader nucleotide context, 5’-UCCGUGUG, surrounding the edited cytosine which could function as a predictor of A3B catalyzed RNA editing sites in the liver and pancreas of these mice (Fig. 4A). In addition, no predicted stem-loop structures with the reactive C at the 3′-end of the loop were found in these regions in contrast to prior reports for A3A RNA editing hotspots [18, 50, 51]. The majority of the C-to-U modifications occurred at 3’UTRs, while 30% of the C-to-U sites were in coding exons, causing 1 stop, 13 non-synonymous and 23 synonymous changes (Fig. 4B). Next, to assess the potential correlation between the recurrent edited transcripts and their corresponding mRNA expression, we compared the expression levels of the recurrent edited positions between A3B samples and controls. While we did not see significant changes in the liver transcripts, the recurrent editing transcripts in the pancreas had higher expression levels than their non-edited counterparts (Fig. 4C). Notably, these editing events predominantly occurred within the 3’UTR, implying that such editing might influence the stability of the edited transcripts. Further studies should focus on studying whether A3B editing may indeed have an impact in biological functions.

Extensive analysis from the RNA edited positions revealed that 46 edited sites were recurrent in the liver from different samples, while 71 sites in the pancreas occurred across different mice (Additional file 1: Fig. S5A, B). More interestingly, we found that 65 positions were shared between the liver and the pancreas (Additional file 1: Fig. S5C). Altogether, these results reinforce our finding that A3B has RNA editing activity in a UCC-specific context which could be interpreted as epigenetic hotspot.

### Endogenous APOBEC enzymes are not responsible for the C-to-U edits in *A3B* mice

**Fig. 5:**
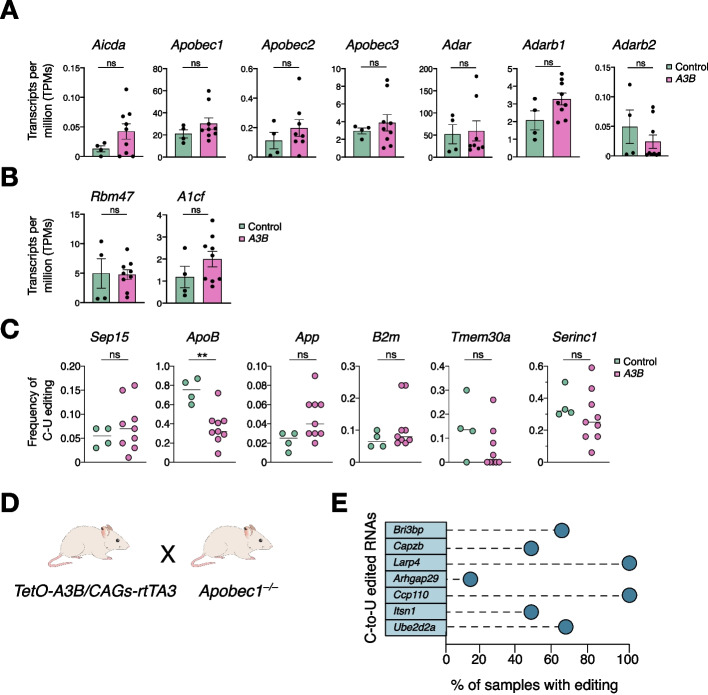
Endogenous Apobec enzymes are not responsible for the observed RNA editing. **A** Average expression levels of the different endogenous *Apobec* and *Adar* family members in liver samples obtained from RNA seq data (transcripts per million (TPMs); each dot represents data from one animal *n* = 4 controls and 9 *A3B* livers). **B** Average expression levels of *Apobec1* cofactors obtained from the RNA seq data and shown as transcripts per million (TPMs); each dot represents data from one animal (*n* = 4 controls and 9 *A3B* livers). **C** Frequency of C-to-U mRNA editing in well-known *Apobec1* editing sites measured by quantification of RNA seq data from controls and *A3B* livers. Each dot represents data from one animal (*n* = 4 controls and 9 *A3B* livers). **D**) Schematic of the breeding strategy to obtain *A3B/Apobec1*^*−/−*^ mice. **E** Lollipop plots indicating the percentage of mice showing C-to-U editing after experimental validation by RT-PCR in selected targets in *A3B/Apobec1*.^*−/−*^ mice (*n* = 6 liver tissues)

The APOBEC family members in rodents include *Apobec1, Apobec2, Apobec3*, and *Aicda* (AID). To address, whether these other enzymes could be responsible for the editing events, we assessed the endogenous expression levels of the different *Apobec* and *Adar* genes (*Aicda*, *Apobec1*, *Apobec2*, *Apobec3*, *Adar*, *Adarb1*, and *Adarb2*) in liver specimens using RNA-seq data. Expression levels of these genes were similar between controls and A3B overexpressing samples, making it unlikely that the RNA editing events were due to the activity of one of these enzymes (Fig. 5A).

Apobec1 is an established RNA editing enzyme and the only family member that shows C-to-U RNA editing activity in mice, being highly active in the liver and the intestine [15, 52, 53]. As *Apobec1* mRNA expression levels were unchanged, we then evaluated whether A3B overexpression might interfere with endogenous Apobec1 functionality and cause the observed editing. A limiting step in RNA editing by Apobec1 is the formation of the editosome complex which requires the binding of Apobec1 to its cofactors: Rbm47 and A1cf. To address this point, we examined the expression of the *Apobec1* cofactors and found that neither *Rbm47* nor *A1cf* expression were changed in *A3B* livers compared to controls (Fig. 5B). Next, we checked well-known RNA editing sites for *Apobec1* [53] and found no significant differences in the editing frequencies from the majority of the *Apobec1* targets, except for the namesake *ApoB* transcript, where editing was reduced by half in *A3B* livers compared to controls (Fig. 5C).

To directly address whether murine APOBEC1 may interact with human A3B and contribute to the observed mRNA editing hotspots in animals overexpressing A3B, *Apobec1* knockout animals [54] were crossed with our A3B inducible model (Fig. 5D). Shortly after doxycycline administration (6–11 days), *Apobec1* knockout animals with inducible human A3B still succumbed to death, demonstrating that the detrimental effect of A3B is independent of murine *Apobec1* (Additional file 1: Fig. S6A). *A3B/Apobec1*^*−/−*^ mice express A3B at lower levels than single *A3B* animals and consistently the deaminase activity of A3B was reduced upon *Apobec1* loss (Additional file 1: Fig. S6B). In addition, the 7 validated and recurrent RNA editing events described in *A3B* livers were still evident in the *A3B/Apobec1*^*−/−*^ samples (Fig. 5E and Additional file 3: Table S2). Altogether, these data show that A3B and not endogenous *Apobec1* is responsible for the observed RNA editing events.

### Continuous expression of catalytically active APOBEC3B is required for RNA editing

**Fig. 6:**
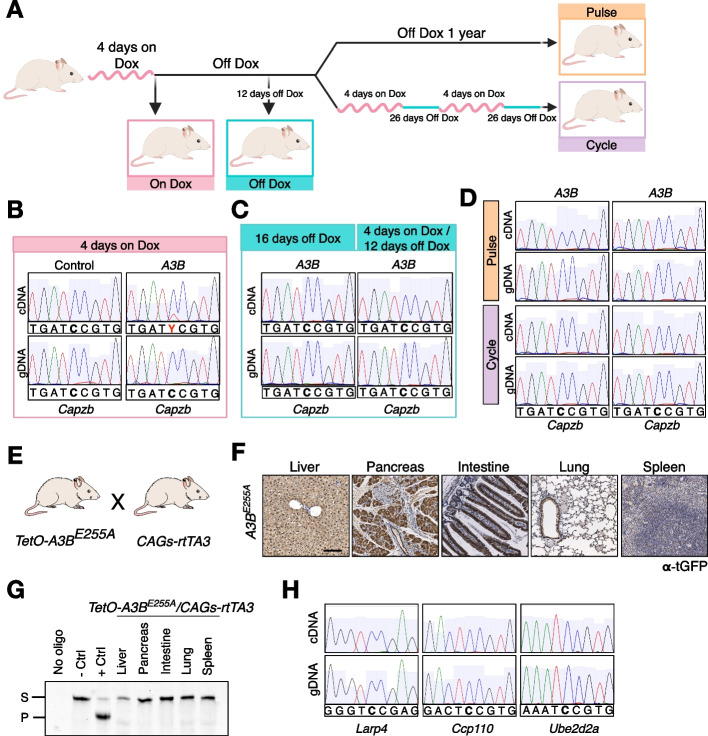
Continuous expression of A3B is required for RNA editing. **A** Schematic representation of the strategy used to study whether A3B expression is needed to detect the RNA edits**. B, C** and** D** Examples of Sanger sequencing chromatograms from the livers for the A3B-driven edited positions. **B** Mice 4 days after dox administration. Control refers to a mouse that has the *TetO-A3B* transgene but not the *CAGs-rtTA3*, while A3B is a mouse with *TetO-A3B/CAGs-rtTA3* genotype. **C** Mice on dox for 4 days and placed back on a normal diet for 12 days or off dox for 16 days. **D** Mice that received a pulse of A3B expression (4 days dox and up to a year on normal diet) or expressing A3B in a cycle manner (4 days dox-26 days off dox/monthly). Samples were collected at experimental endpoint (1 year). **E** Schematic of the breeding strategy to obtain *TetO-A3B-*^*E255A*^*/CAGs-rtTA3* mice. **F** Immunohistochemistry of tGFP in the indicated tissues from *A3B-*^*E255A*^ fed with dox for 8 days. Scale bar: 100 μm. **G** Deamination activity assay in the indicated tissues from *TetO-A3B-*^*E255A*^*/CAGs-rtTA3* mice fed with dox for 8 days (S, Substrate; P, Product). **H** Examples of Sanger sequencing chromatograms showing no RNA editing in *A3B-*^*E255A*^ liver tissues

To understand whether continuous expression of A3B is required to edit the RNA, we took advantage of the doxycycline-inducible model which allows to silence transgene expression after dox withdrawal. Mice were fed with doxycycline for 4 days, followed by doxycycline deprivation to abrogate A3B expression and liver samples were collected at defined time points to further check RNA editing. On the one hand, we analyzed liver samples 4 days after doxycycline administration and 12 days after doxycycline withdrawal. On the other hand, after 12 days off dox, mice received either normal food for1 year (pulse) or expressed A3B in cycles (4 days on dox plus 26 days off dox) every month for 1 year (cycle) (Fig. 6A). We observed that 4 days of doxycycline administration were sufficient to trigger *A3B* expression (Additional file 1: Fig. S7A) and consequently induce A3B-driven RNA editing events in 6 out of the 7 positions examined in *A3B* samples but not in control (Fig. 6B and Additional file 3: Table S2). Importantly, no DNA damage was observed in the livers 4 days after A3B expression, suggesting that RNA editing arises earlier than DNA damage making it unlikely that RNA editing is a consequence of the DNA damage induced by A3B (Additional file 1: Fig. S7B). Moreover, doxycycline withdrawal for 12 days was enough to abrogate A3B expression and subsequently A3B-driven RNA edited positions could no longer be detected (Fig. 6C, Additional file 1: S7C and Additional file 3: Table S2). Accordingly, mice expressing A3B in a pulse or a cycle manner showed no expression of A3B and no detectable RNA editing (Fig. 6D, Additional file 1: S7D and Additional file 3: Table S2). Altogether, these results indicate that continuous expression of *A3B* is required for RNA editing.

To discern whether the RNA editing events in *A3B* mice are a consequence of the deaminase activity, we generated catalytically inactive *A3B* mutant mice by site-directed mutagenesis of one of the A3B catalytic domains (E255A). We followed the same strategy used to generate *A3B* mice and *TetO-A3B*^*E255A*^ animals were crossed with *CAGs-rtTA3* mice (Fig. 6E). Doxycycline administration to *A3B*^*E255A*^/*CAGs-rtTA3* mice resulted in strong expression of catalytically dead A3B in the liver and pancreas, with moderate levels in the intestine, whereas the lung and the spleen contained only modest levels of this protein (Fig. 6F and Additional file 1: S7E,F). As expected, soluble protein extracts from *A3B*^*E255A*^-expressing tissues showed no deaminase activity, in any of the tissues tested (Fig. 6G). To determine whether RNA editing activity was deaminase dependent, recurrent liver RNA editing events were validated in the livers of 3 *A3B*^*E255A*^ mice after 10 days on doxycycline. No C-to-U changes were detected in any of the 7 positions examined, indicating that RNA editing activity by A3B requires a functional deaminase domain (Fig. 6H and Additional file 3: Table S2).

## Discussion

A3B, a single-stranded-DNA C-to-U editing enzyme, is in the spotlight of cancer research as a driver of tumor evolution and therapy resistance. Several studies have attributed A3B-induced tumor heterogeneity to its DNA mutagenic activity [25, 55–57]. Moreover, a recent study has shown that A3B-induced DNA damage can also contribute to chromosomal instability and increased tumor heterogeneity [58]. However, whether A3B can also influence tumorigenesis by RNA editing activity remains unknown. Employing a novel doxycycline inducible mouse model, in which high and sustained A3B levels were achieved, we found that A3B is not well tolerated by cells and it compromises animal survival by inducing DNA damage and inflammation. In addition, we show that A3B catalyzes C-to-U deamination in mRNA in a catalytic-dependent manner, as evidenced by a complete loss of mRNA editing hotspots in *A3B*^*E255A*^ expressing animals. A3B was previously described to solely deaminate ssDNA cytosines and, therefore, this is the first demonstration in vivo that human A3B has the capacity to edit RNA cytosines and change the epigenome.

APOBEC1 and APOBEC3A have a dual function in editing RNA and inducing mutations in DNA [59]. The RNA editing functions of these enzymes are in part explained by sequence motifs. For instance, APOBEC1-edited cytosines often occur adjacent to an AU-rich motif, which is postulated to be a binding sequence for a larger editosome complex [5, 59]. In comparison, A3A has a strong preference for RNA cytosines at the 3’ side of the loop region of stem-loop structures [18, 51]. Our study here shows that the most frequently edited trinucleotide in RNA in *A3B* expressing mice is UCC-to-UUC. A structural explanation for this difference is unrelated to cruciform/hairpin structures. However, one clue may derive from the broader sequence motif of A3B hotspots, UCCGUGUG. Prior studies have demonstrated that repeated GU dinucleotide motifs in RNA are capable of forming G4 (G-quartet) structures with the U-nucleobases extruded [60]. Adjacent UCC motifs are therefore likely to be single-stranded and potentially exposed to A3B for C-to-U editing. Structural studies will be necessary to unambiguously test this mechanistic possibility.

It has been reported that continuous expression of high levels of A3A and A3B can be toxic to cells [25, 36, 61]. Similarly, acute and sustained expression of A3B in the inducible mouse model described here disrupts tissue homeostasis and causes lethality. Interestingly, genetic ablation of *Apobec1* increased the severity of the phenotype of *A3B*-expressing mice. Although Apobec1 deficiency does not cause any abnormalities in the mice in short term [54], these findings suggest that the detrimental effect of A3B is “enhanced” upon the loss of murine *Apobec1*. It has been reported that aged *Apobec1* deficient mice exhibit elevated atherosclerosis levels [54, 62]. Moreover, differential expression analysis of A3B-expressing livers revealed a significant downregulation in fatty acid metabolism. Therefore, the accelerated lethality could be the result of a catastrophic failure in lipid metabolism. Although the A3B levels achieved in our model are not compatible with the survival of these animals, they recapitulate the levels found in a subset of human cancers. It is not clear why human tumor cells tolerate similarly high levels of A3B, whereas the animals here do not, but it may relate to the development of a tolerance mechanism that involves *TP53* inactivation [25].

## Conclusions

We report here, for the first time that A3B, a known genome mutagenic enzyme, is also able to deaminate the RNA in mice, when overexpressed. We discovered that A3B-associated edits occur mainly at the UCC motif and that its expression and deaminase activity are required to detect the edits. This evidence and the emerging collective findings in other family members suggest that the APOBEC family may utilize a combination of different mechanisms to induce genetic variability. These results highlight the importance of expanding our knowledge of C-to-U RNA-editing events. Because of the dynamic nature of RNA editing, it will be important to identify the timing when A3B is upregulated in human tumors to fully evaluate its implications.

## Materials & methods

### Mouse models

KH2 ES cells were a gift from Sagrario Ortega and were generated by Konrad Hochedlinger and Rudolf Jaenisch [41]. These ES cells carry the M2-rtTA gene inserted within the Rosa26 allele. A construct containing the human *APOBEC3B-tGFP* cDNA (OriGene, NM_004900) or the *APOBEC3B-E255A-tGFP* cDNA under the control of the tetracycline response element (TRE) was inserted downstream of the *Col1A1* locus. *ColA1-APOBEC3B*/*Rosa26-rtTA* and *ColA1-APOBEC3B-E255A*/*Rosa26-rtTA* heterozygous animals were bred out to exclude the *Rosa26-rtTA* transgene and bred to *CAGs-rtTA3* mice [42]. All mice used in this study are included in Additional file 4: Table S3.

For inducing the transgene in vivo, different genotypes of the APOBEC3B:rtTA alleles [ (+ */A3B*)(+ */rtTA*); (+ / +)(+ /*rtTA*); (+ /*A3B*)(+ */* +)] were fed with 625 ppm dox impregnated food pellets. For control, littermates with the genotypes (+ / +)(+ /*rtTA*); (+ /*A3B*)(+ / +) were fed with doxycycline. For the mutant mice, only this genotype was used (+ */A3B*^*E255A*^)(+ */rtTA*).

Isolation of ear punch-DNA was performed via incubation in 100µL 0.05 M NaOH at 98ºC for 1 h and subsequent neutralization with 10µL 1 M Tris HCl ph7.5. *Rosa26-rtTA and CAGs-rtTA* transgenic mice were genotyped as described previously [42]. The following oligonucleotides were used to genotype the *ColA1-A3B and ColA1-A3B*^*E255A*^ alleles: KH2-A3B A: 5’GCTGGGACACCTTTGTGTACCG 3’, KH2-A3B B: 5’ATCACGTGGCTCAGCAGGTAGG 3’. For all transgenes, the following PCR program was applied: 94 °C for 2 min, 30 times [95 °C for 30 s, 60 °C for 30 s, 72 °C for 30 s], and a final step at 72 °C for 1 min.

### Generation of APOBEC3B-E255A cDNA

For generating the A3B-E255A-tGFP plasmid, site-directed mutagenesis was performed by using Q5 site-directed mutagenesis kit (NEB) and following the manufacturer’s instructions. Primers were designed to create targeted specific changes (E255A, adenine for cytosine in position 255) in the plasmid containing the wild-type A3B. This plasmid was used for electroporating the ES cells to generate mutant *A3B*^*E255A*^ transgenic mice, as previously described.

### Immunodetection in tissue sections

Tissues were fixed in formalin overnight and embedded in paraffin. Antigen retrieval was performed using 0.09% (v/v) unmasking solution (Vector Labs, H-3300) for 30 min in a steamer. Inactivation of endogenous peroxidases was done using 3% Hydrogen Peroxide (Sigma, H1009) for 10 min. Secondary antibody staining and biotin-streptavidin incubation were performed using species-specific VECTASTAIN Elite ABC kits (Vector Labs, PK-6101 and BMK-2202). DAB Peroxidase Substrate kit (Vector Labs, SK-4100) was utilized for antibody detection. Primary antibodies used were anti-pH3 Ser10 (1:200, Cell Signalling, 9701), cleaved caspase 3 (1:200, Cell Signalling, 9661), γH2AX (1:200, Bethyl Labs 00059), APOBEC3B (5210–87-13 mAb, 1:200) [63], tGFP (1:200 Origene, TA150041), Ki67 (Medac 275R-18). Sections were visualized under a TissueFAXS slide scanning platform (TissueGnostics, Vienna, Austria). All the quantifications were done using StrataQuest software (TissueGnostics) to determine the number or percentage of pH3, Casp3, ki67 and γH2AX. Eosin G was from Roth and hematoxylin was from Linaris.

### Measurement of serum parameters

The analysis for AST, ALT, lipase and amylase was performed with mouse serum with the DRY-CHEM 500i analyzer (Fujifilm, Japan) following the manufacturer’s protocol.

### Deamination assay

DNA deaminase activity was measured in whole lysates of different tissues using previously described protocols [44]. A Fluor-labelled oligonucleotide containing a single target cytosine (5′-ATTATTATTATTCGAATGGATTTATTTATTTATTTATTTATTT-fluorescein) was incubated 3 h at 37 °C with the tissue lysates containing or not A3B. Samples were run in a 15% denaturing acrylamide gel and deamination activity was detected by fluorescence using iBright CL1500 imaging system (Thermo Fisher Scientific). All quantifications were done using Fiji Software to determine the percentage of the deaminated oligo.

### Western blot assay

For protein extraction and immunoblotting, mouse tissues were lysed in RIPA lysis buffer (0.25 M Tris–HCl pH 6.8, 2.5% glycerol, 1% SDS, and 50 mM DTT). Samples were then boiled for 10 min and cleared by centrifugation. Protein expression was assessed by immunoblotting using 40–90 μg of the lysates and probed using the following specific antibodies: anti-APOBEC3B (5210–87-13 mAb, 1:1000) [63]; anti-tGFP (1:1000 Origene, TA150041) GAPDH (1:2000 Millipore, CB1001) and anti-actin (1:5000 Sigma, A2066). Protein detection was done using iBright CL1500 imaging system (Thermo Fisher Scientific) and further quantifications using Fiji Software to determine the amount of protein relative to the loading control.

### DNA and RNA isolation

Snap-frozen tissues were ground with a mortar and pestle on dry ice. For total RNA and genomic DNA extraction, AllPrep DNA/RNA Mini (Qiagen, 80,204) was used and cDNA synthesis was done using the QuantiTect Reverse Transcription Kit (Qiagen, 205,313) according to the manufacturer’s instructions.

### Real-time PCR

Quantification using real-time PCR was initiated using 10 ng of cDNA with SYBR Green PCR Master Mix (2 ×) (Applied Biosystems, 4,364,346) in a LightCycler II®480 (Roche) Relative mRNA levels were calculated according to the ΔCt or ΔΔCt relative quantification method and were normalized to the examined house-keeping genes (18S; Actin; TBP) levels.

### RNA-seq and WES data processing

A3B-dependent RNA editing events in mouse pancreas and liver tissues were identified from matched RNA-seq and WES data of individual A3B mice and a pool of control littermates.

WES libraries were prepared from 0.5 μg of genomic DNA using Agilent Low Input Exom-Seq Mouse kit for Illumina platforms and sequenced with Illumina HiSeq 2000 v4 technology (100-nucleotide paired-end reads). WES data were aligned to the mouse reference genome assembly (mm10) using SpeedSeq [64]. PCR duplicates were removed using Picard tools (version 2.18.16). Reads were locally realigned around Indels using GATK3 (version 3.6.0) tools. Single base substitutions (SBS) in tissues from A3B-overexpressing animals were called relative to the pool of WES data of normal tissues from control littermates using Mutect2. SBSs that passed the internal GATK3 filter with a minimum of 4 reads supporting each variant, minimum 20 total reads at each variant site and a variant allele frequency larger than 0.05 were used for downstream analysis.

Bulk RNA-seq libraries were prepared from 1.2 μg of total RNA with TruSeq Stranded kit for Illumina platforms and sequenced with Illumina HiSeq 2000 v4 technology (100-nucleotide paired-end reads). Before and after trimming of the RNA-seq data, the RNAseq quality was evaluated using FastQC (https://www.bioinformatics.babraham.ac.uk/projects/fastqc/). Quality control, including per-base quality, duplication levels, and over-representative sequences, passed all the checkpoints. RNA-seq reads were aligned to mouse reference genome assembly mm10 using STAR/2.7.1a with basic two pass mode for realigning splice junctions enabled. Picard tools (version 2.18.16) were then used to mark duplicate reads and split CIGAR reads with Ns at the splice junctions. Mutect2 from GATK (3.6) was used to call RNA variants in tissues from A3B-overexpressing animals relative to the pool of normal RNA-seq data from normal tissues of control littermates. RNA variants that passed Mutect2 internal filter with at least 6 reads supporting the variant, a minimum of 20 total reads at the altered site and a variant allele frequency greater than 0.05 were used for downstream analysis. RNA editing levels at Apobec1 target sites were extracted from the raw tables of the aligned reads before applying any filters for further analysis.

Variants detected at the RNA level were considered an RNA editing event when the same variant was not present on the DNA level in the matched exome data from each sample and used for subsequent signature analysis. Relative contribution refers to the contribution that each single base substitution has to the overall base substitution spectrum in A3B mice. All sequence logos were generated and visualized using ggseqlogo package in R. Full context RNA editing events were displayed using PlotRNAedits (https://github.com/temizna/plotRNAedits).

For differential expression analysis on the RNA-seq data, the sequenced reads were aligned to the mouse reference genome assembly mm10 using kallisto v0.46.1. Raw counts were normalized and differentially expressed genes (DEGs) were calculated using the DEseq2 package in R. Gene set enrichment analysis (GSEA) of differentially expressed genes was performed using ‘ClusterProfiler’ package of R and javaGSEA Desktop Application v2.2.2.

Pathways were considered significantly enriched at an FDR equal to or smaller than 0.25.

TPMs levels of various genes were extracted by generating a read-count matrix with the Bioconductor packages GenomicAlignments and GenomicFeatures in R.

All sequences logos were generated and visualized using “ggseqlogo” package in R. Full context RNA editing evens were displayed using PlotRNAedits (https://github.com/temizna/plotRNAedits).

### Candidate validation

Candidates were selected following the criteria that 20% of the transcripts for a specific gene must contain a C-to-T change, to ensure the visualization of a double peak after sanger sequencing. Primers were designed (Additional file 5: Table S4) for the chosen candidates and used to amplify the edited position in RNA and DNA from livers, pancreas and lungs. PCR products were run in a 1% agarose gel and later bands were isolated and DNA extracted using QIAquick Gel Extraction kit (Qiagen). The extracted DNA was then submitted for sanger sequencing (Microsynth). Sequences were aligned to the specific reference mouse gene using the online tool Benchling to finally determine the presence of an RNA editing event or a DNA mutation. RNA editing events were additionally verified and quantified using MultiEditR v1.0.8 [65].

## Statistical analysis

Statistical analysis was carried out using Prism6 (GraphPad). *p* values were as follows: *p < 0.05, **p < 0.01, ***p < 0.001, ****p < 0.0001. The number of animals is represented with n.

### Supplementary Information

**Additional file 1.** Supplementary figures.

**Additional file 2:** **Table S1.** RNA editing events per chromosome.

**Additional file 3:** **Table S2.** RNA editing validation.

**Additional file 4:** **Table S3.** Mice.

**Additional file 5:** **Table S4.** Sequences of primers used for the validation.

**Additional file 6.** Data source.

**Additional file 7.** Peer review history.

## Data Availability

All data generated in this study is available at NCBI Gene expression Omnibus (GEO) https://www.ncbi.nlm.nih.gov/geo/query/acc.cgi?acc=GSE209723 under accession number GSE209723 [66].

## Abbreviations

A3A: APOBEC3A
A3B: APOBEC3B
A3G: APOBEC3G
SBS: Single base substitution
Dox: Doxycycline
TBP: TATA-binding protein
RNA-seq: RNA-sequencing
ALT: Alanine transaminase
AST: Aspartate transaminase
WES: Whole exome sequencing
SNPs: Single nucleotide polymorphism
