# Supplementary material for: Acute expression of human APOBEC3B in mice results in RNA editing and lethality
Source: Genome Biol. 2023 Nov 24;24:267. doi: 10.1186/s13059-023-03115-4 (PMC10668425; doi:10.1186/s13059-023-03115-4)
Supplement: Supplementary file 1 — Additional file 1. Supplementary figures. [file 13059_2023_3115_MOESM1_ESM.pdf]

Supplementary Text and Figures

Acute expression of human APOBEC3B in mice results in RNA editing and lethality

Supplementary Figure Legends

Fig S1.

A) Schematic of the breeding strategy to obtain *TetO-A3B/CAGs-rtTA3* mice. **B**) Quantitative RT-PCR analysis of A3B transgene expression in different tissues from *A3B* mice after 10 days on doxycycline normalized to two housekeeping genes: Actin and 18S. Error bars represent SEM. Each dot is a mouse. **C**) Immunohistochemistry of A3B in the indicated tissues from *TetO-A3B/*CAGs-rtTA3 mice fed with doxycycline for 10 days. Scale bar: 50 µm. **D**) DNA cytosine deaminase activity with increasing amounts of protein extracts from liver tissue and their corresponding quantification (n=4) (S, Substrate; P, Product). **E**) A3B mRNA levels in liver, lung and pancreas from *TetO-A3B/CAGs-rtTA3* animals are within the range reported for human tumors (gray dots represent data from individual TCGA tumors, purple dots represent liver, green dots pancreas and yellow dots lung tissues from *TetO-A3B/CAGs-rtTA3* mice; mean ± SD shown by red dots and error bars, respectively).

Fig. S2.

A) Immunohistochemistry against pH3 and ki67 in paraffin sections of livers from control and *A3B* mice and their corresponding quantification (n=6). **B**) Pathway enrichment analysis showing the normalized enrichment score (NES) for the *A3B* liver samples compared to the controls. False discovery rate (FDR) q-values are shown to the right or left side of the bars. **C**) Analysis of transaminase levels (ALT and AST), lipase and amylase in sera of control and *A3B* mice. **D**) Pathway enrichment analysis showing the normalized enrichment score (NES) for the *A3B* pancreas samples compared to the controls. False discovery rate (FDR) q-values are shown on the right or left side of the bars. **E**) Immunohistochemistry against pH3 and ki67 in paraffin sections of pancreas from control and *A3B* mice and their corresponding quantification (n=6). Data was analyzed by unpaired t test, n.s. non-significant. Data represented as mean ± SD are shown by dots and error bars, respectively. Scale bar: 100 µm.

Fig. S3.

A) Trinucleotide mutation profiles for all base substitutions at the RNA level in livers from each individual *A3B* mouse. **B**) Trinucleotide mutation profiles for all base substitutions in

pancreas from each individual *A3B* mouse. Relative contribution refers to the contribution that each single base substitution has to the overall base substitution spectrum in A3B mice.

Fig. S4.

A) Trinucleotide mutation profiles for all base substitutions at the DNA level in livers and pancreas from *A3B* mice, n=6. Relative contribution refers to the contribution that each single base substitution has to the overall base substitution spectrum in A3B mice. **B**) and **C)** Examples of Sanger sequencing chromatograms showing A3B-driven RNA editing in *A3B* liver and pancreatic tissues, respectively. **D**) A3B mRNA levels in liver and lung from individual *A3B* mice relative to TBP (mean ± SEM shown by dots and error bars, respectively) Unpaired t-test, p<0,001. **E**) Examples of Sanger sequencing chromatograms showing A3B-driven DNA mutations in *A3B* lungs.

Fig. S5.

Editing landscape of shared edited genes in A3B-expressing livers and pancreas. The upper panel indicates the total number of edits per mouse and the lower panel indicates the shared edits among mice. Represented only those edits that appeared in 2 or more mice. **A**) Liver **B**) Pancreas **C**) Liver and pancreas shared edits.

Fig. S6.

A) Survival of *A3B/CAGs-rtTA3* and *A3B/Apobec1$^{-/-}$/CAGs-rtTA3* mice after doxycycline administration. *A3B* n= 26, *A3B/Apobec1-/-* n=6. P<0.0001 by Log-rank (Mantel-Cox) test. **B**) Immunodetection of tGFP in the indicated tissues from *A3B* and *A3B/Apobec1$^{-/-}$* mice fed with doxycycline for 10 and 7 days respectively, Anti-actin blots are shown as loading controls. Below, the corresponding quantification. **C**) DNA cytosine deaminase activity from whole-cell extracts in the indicated tissues from *A3B* and *A3B/Apobec1$^{-/-}$* mice and the corresponding quantification of the percentage of deaminated oligo (S, substrate; P, product).

Fig. S7.

A) Western blot showing tGFP levels in liver tissue from *A3B* mice 4 days after dox administration. Actin is shown as the loading control. **B**) Immunohistochemistry against gH2AX in paraffin sections of livers from *A3B* mice 4 and 10 days after doxycycline administration. **C**) Western blot showing tGFP levels in liver tissue from control and *A3B* mice

on dox for 4 days and placed back on a normal diet for 12 days. Actin is shown as the loading control. **D**) Western blot showing tGFP levels in liver tissue from control and *A3B* mice that received a pulse of A3B expression (4 days dox and up to a year on a normal diet) or expressing A3B in a cycle manner (4 days dox-26 days off dox/monthly). Samples were collected at experimental endpoint (1 year). GAPDH is shown as the loading control and the quantification is shown on the right. **E**) Immunoblot showing A3B-tGFP levels in the indicated tissues from $A3B^{E255A}$ mice fed with dox for 8 days. GAPDH or actin were used as the loading control. On the right, corresponding quantification. **F**) Western blot showing tGFP levels in liver tissue of 3 *A3B* and 3 $A3B^{\mathrm{E255A}}$ and the corresponding quantification. GAPDH is shown as the loading control.

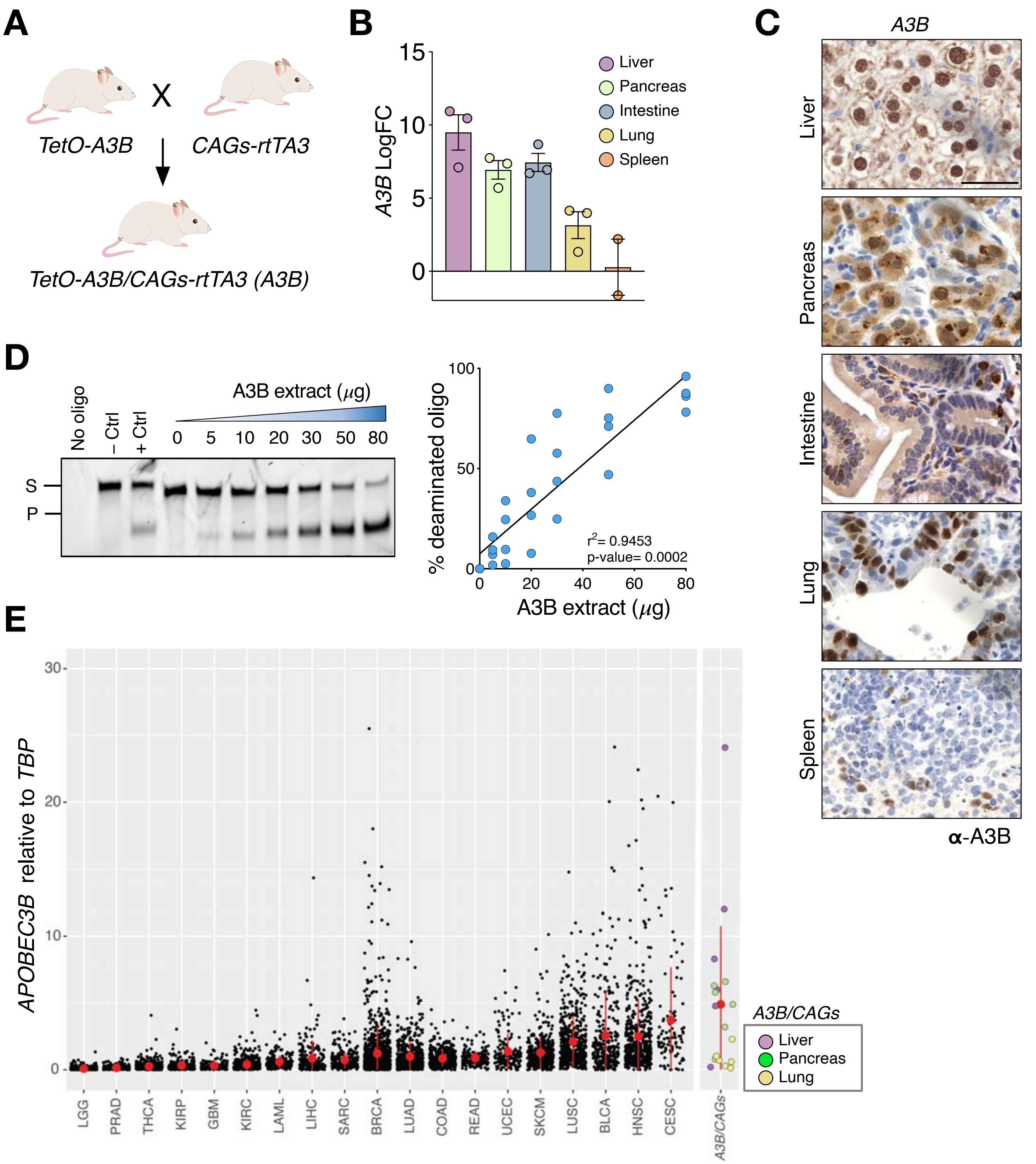

Supplemental Figure 1

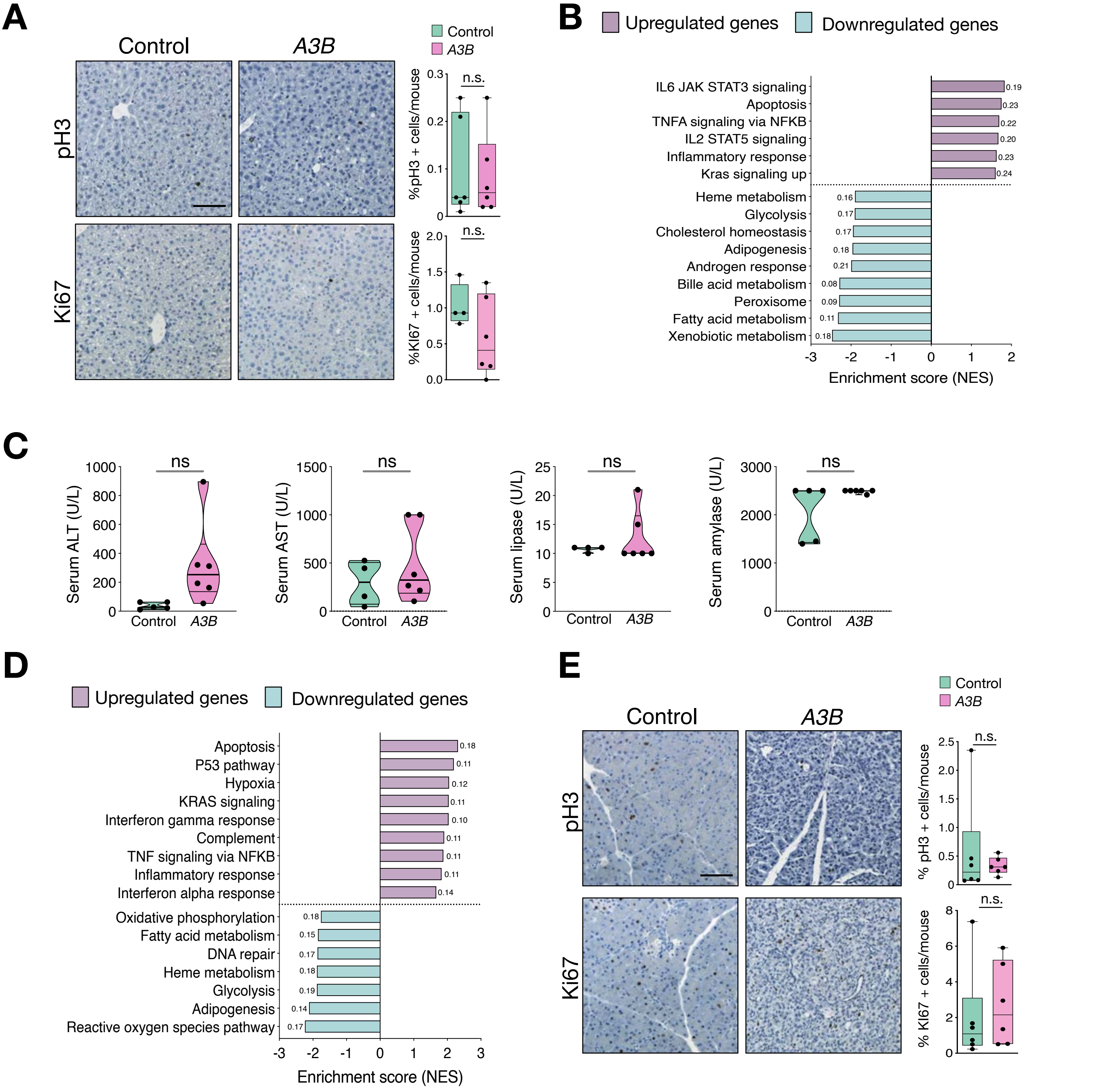

Supplemental Figure 2

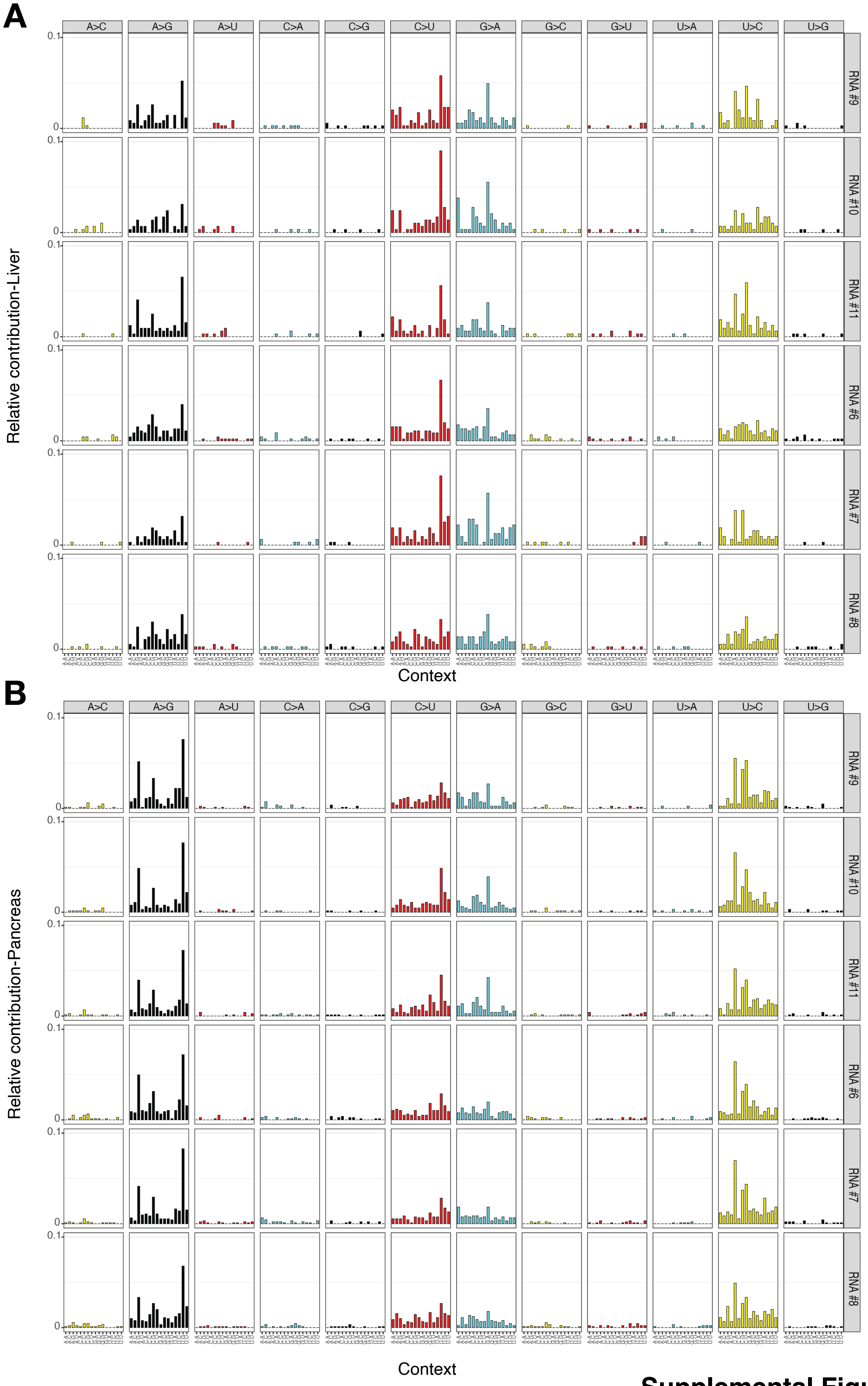

Supplemental Figure 3

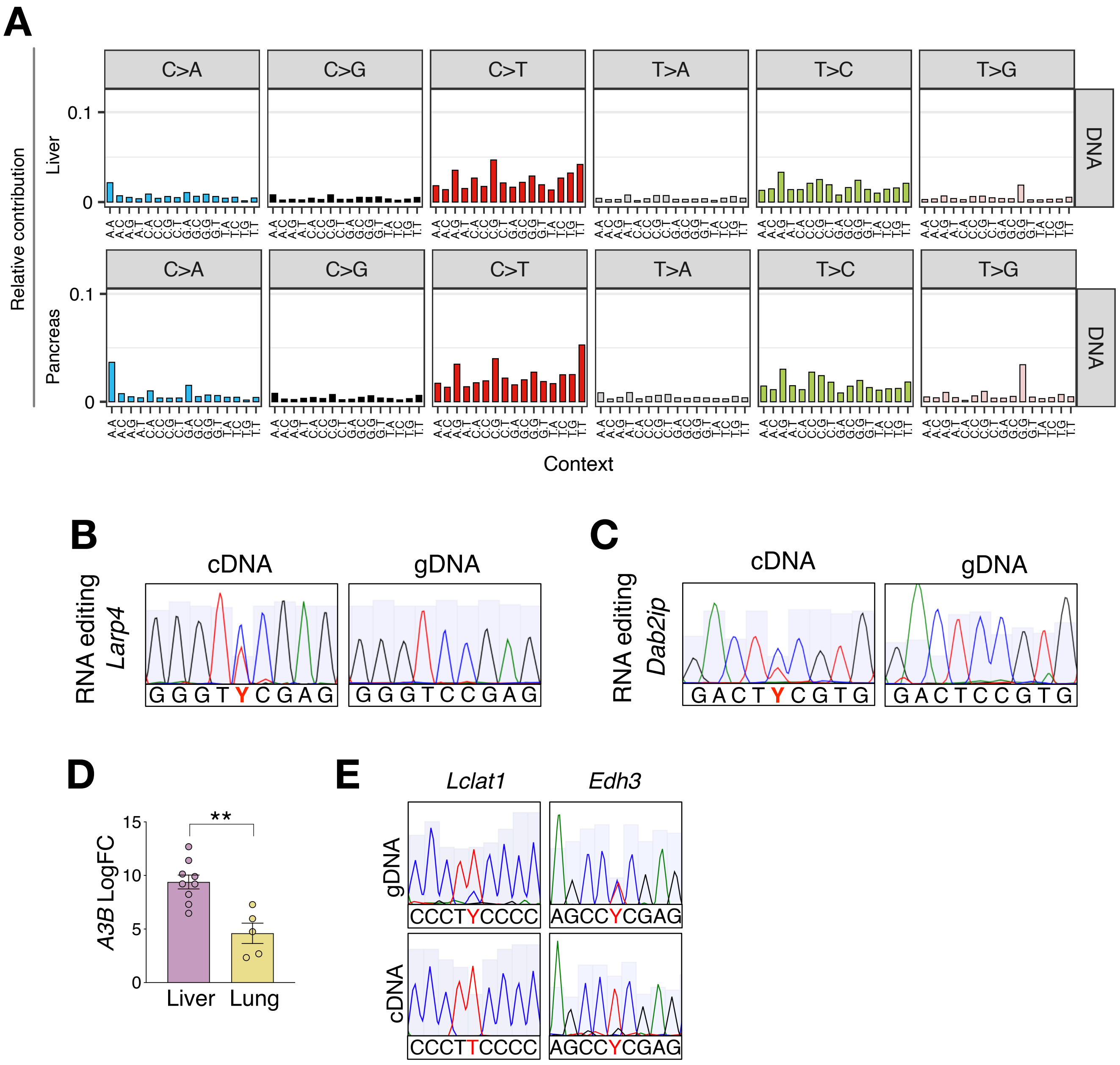

Supplemental Figure 4

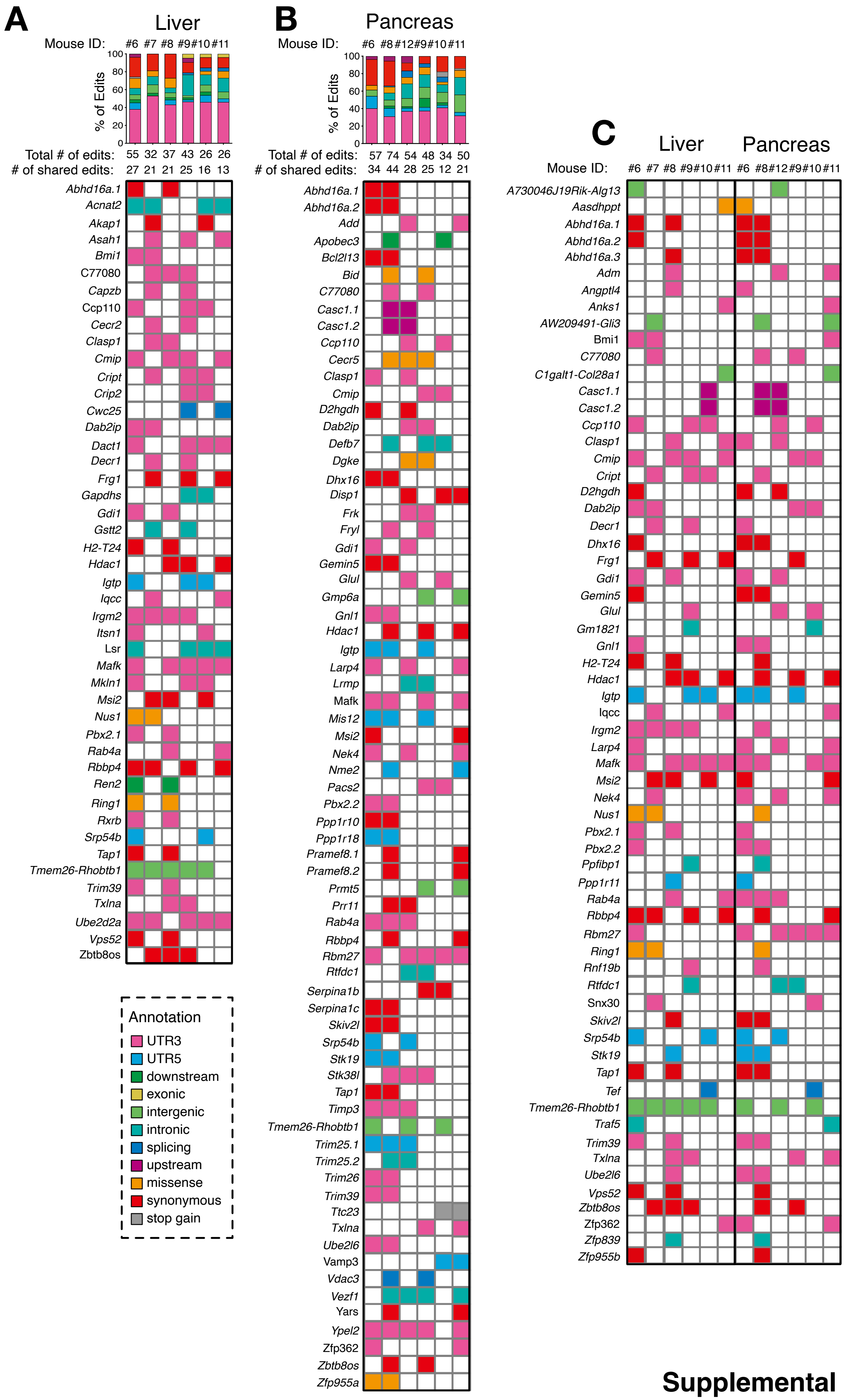

Supplemental Figure 5

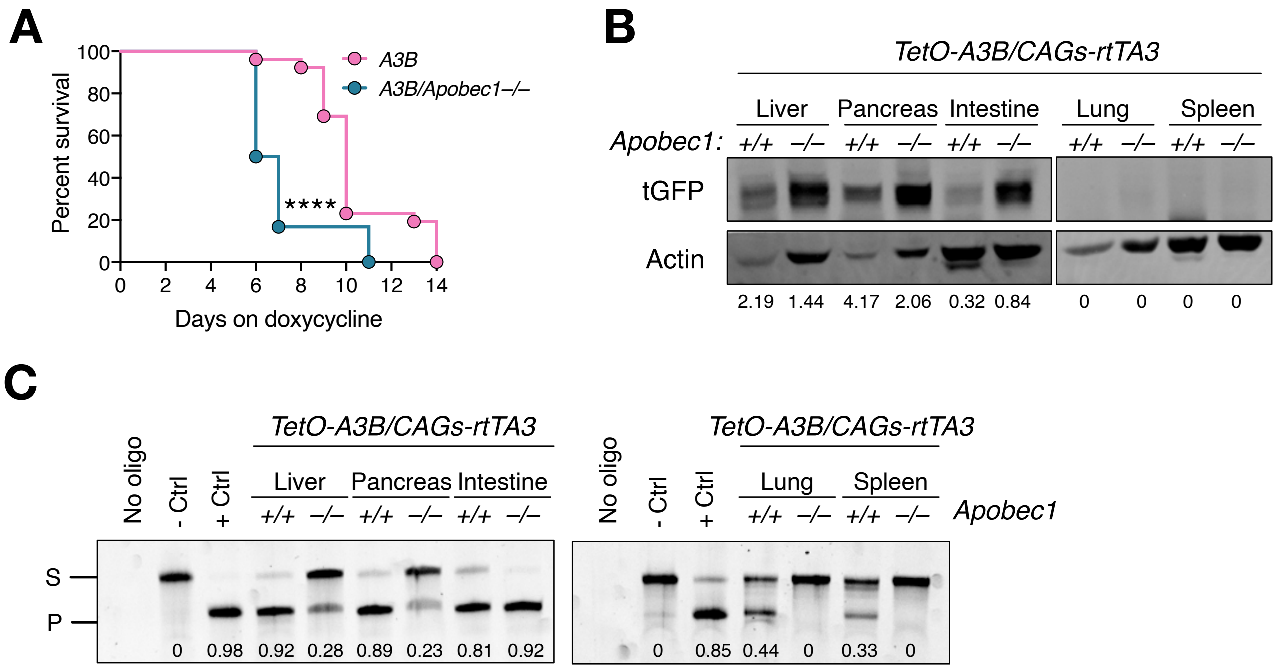

Supplemental Figure 6

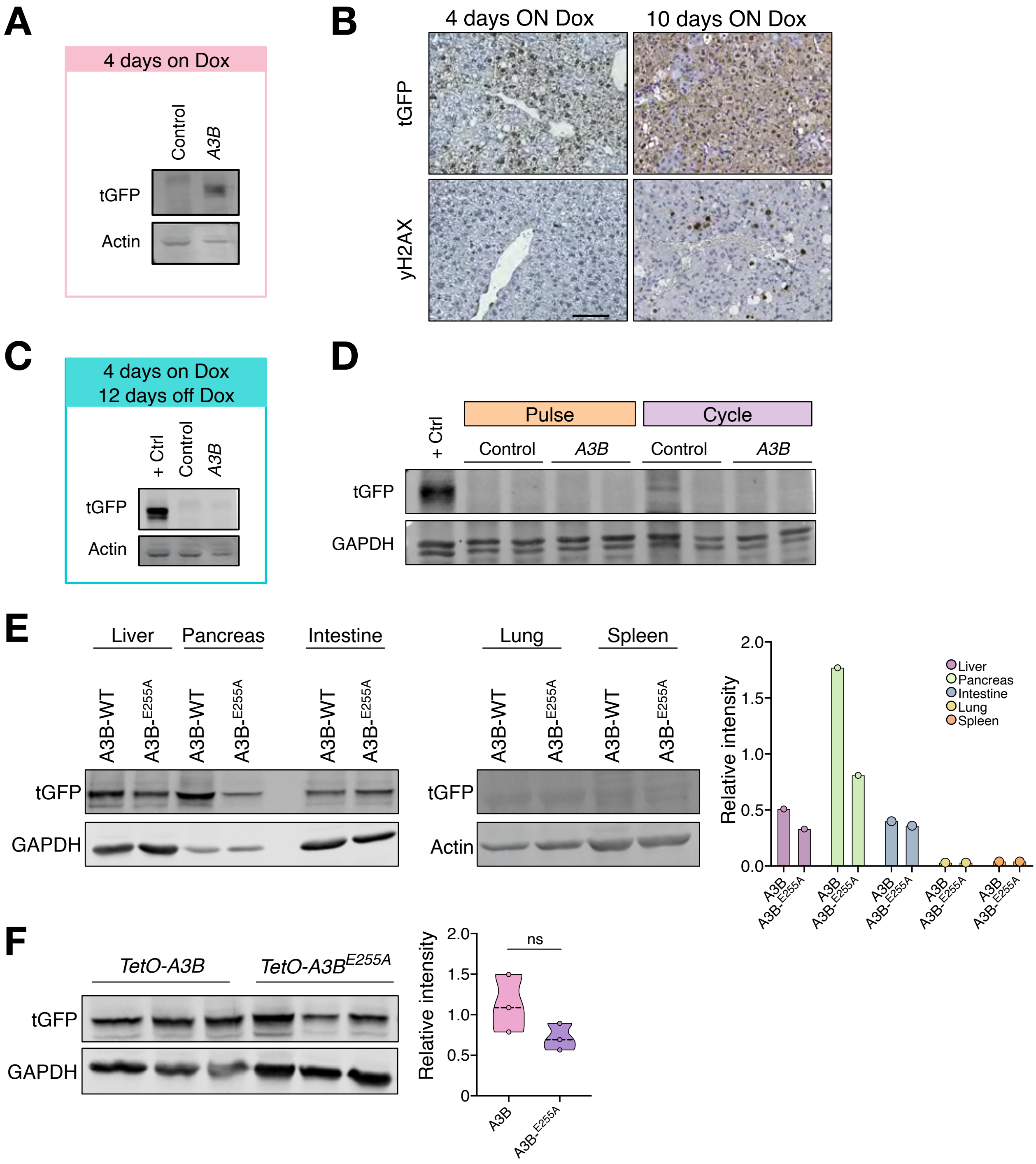

Supplemental Figure 7
