# Supplementary material for: Acute expression of human APOBEC3B in mice results in RNA editing and lethality
Source: Genome Biol. 2023 Nov 24;24:267. doi: 10.1186/s13059-023-03115-4 (PMC10668425; doi:10.1186/s13059-023-03115-4)
Supplement: Supplementary file 7 — Additional file 7. Peer review history. [file 13059_2023_3115_MOESM7_ESM.docx]

**Review history**

**First round of review**

**Reviewer 1**

The manuscript Acute expression of human APOBEC3B in mice results in RNA editing and lethality demonstrates for the first time experimentally that overexpression of human A3B is lethal in an animal model. Further, this is the first study to show that A3B functions as an RNA deaminase. Overall the data are convincing, interesting, and novel. While the study is largely descriptive/phenotypic, it provides a foundation for further research in the field to determine the mechanism of animal death from A3B overexpression and also the effect of RNA editing on the animal. The data spur interesting biochemical questions about the RNA substrate preference of A3B. This is a well-written and timely manuscript which has my support for publication with a few minor suggestions listed below.

RNA editing is not intuitively associated with driving cancer or other diseases. It would be nice to have a hypothesis for this mechanism stated in the introduction, to set the stage for subsequent data and discussion.
Line 29-32: RNA editing is a dynamic process, but the RNA edits don't necessarily disappear right after the responsible enzyme is gone. Loss of RNA edits also requires turnover of transcripts. I suggest a clarification of this statement.
Supp Fig 1E - the dots representing A3B levels on the far right are hard to see and colors are not discernable.
Fig 3B - please clarify in the legend and methods how relative contribution is defined. Is this the absolute amount of RNA edits in A3B mice? Or is it measured relative to control mice?
Supp Fig 4 - the color coding is a little confusing because it encompasses both location and type of mutation but makes them mutually exclusive. These categories could co-occur (for example, a missense mutation in the 3'UTR) but the table does not allow for that information to be conveyed.

**Reviewer 2**

The manuscript by Alonso de la Vega and Colleagues reports the effects of APOBEC3B artificial induction in transgenic mice. The mice were generated by introducing an expression cassette bearing the coding sequence for an APOBEC3B-turboGFP chimera into the ColA1 locus under the control of a TetOn inducible promoter. The experiments were carried out after induction of the APOBEC3B chimera in 4-weeks old mice through doxycycline feeding.
The expression levels of the chimera were compared to the physiological/pathological ones in human tissues to make sure these were in the same order of magnitude.
The two main findings are:
- prolonged expression of the APOBEC3B is lethal in mice
- with very high levels of APOBEC3B, its ability to target RNA becomes evident.
Overall, the manuscript is quite interesting: as it asks questions that have been lingering around for a while - specifically, how comparable are APOBEC3A and APOBEC3B, as they share a virtually identical C-terminal domain? While a more exhaustive answer would have been preferable, the short answer is probably good enough: the two proteins are probably more similar than we would like to acknowledge, with the higher genotoxicity of APOBEC3B being the reason for these differences (APOBEC3B inability to trigger tumors in murine models and the failure to detect APOBEC3B dependent RNA editing in human cells).

Major comments
- As it might be relevant for DNA accessibility, it would be useful to show that localization of the APOBEC3B-turboGFP chimera is the same as that of wild-type APOBEC3B (and how it compares to APOBEC3A).
- How similar is the phenotype of APOBEC3B expressing mice to those from other genotoxic agents? If specific tissues/pathways that are responsible for the phenotype were identified for other genotoxic agents, it might be worth assessing them also in these mice.
- Considering that RNA-seq experiments were performed using stranded libraries, there is no reason to group together C>U/G>A and A>G/U>C changes. This is quite important, as G>A and U>C changes can be used as internal controls for RNA editing. A useful way to depict the specificity of the RNA editing could be to plot each position with a change through its (average) frequency and its recurrence: only C>U and A>G changes should present a bias.

Minor points
- Is there any correlation between RNA editing levels and the expression levels of the edited transcripts - especially in the case of the recurrent sites?
- It would be useful to add the editing frequency in Table 2 as well
- It would be useful if the Authors could discuss how they explain the specificity/frequency of the editing at some of the sites with the requirement for a very high APOBEC3B expression. I would imagine that, with such specificity/frequency, even lower APOBEC3B expression would be sufficient to detect the editing.
- page 6. "Feed ab libitum" should be "fed ad libitum"

**Authors’ response to reviewers**

**Reviewer #1**: The manuscript Acute expression of human APOBEC3B in mice results in RNA editing and lethality demonstrates for the first time experimentally that overexpression of human A3B is lethal in an animal model. Further, this is the first study to show that A3B functions as an RNA deaminase. Overall, the data are convincing, interesting, and novel. While the study is largely descriptive/phenotypic, it provides a foundation for further research in the field to determine the mechanism of animal death from A3B overexpression and also the effect of RNA editing on the animal. The data spur interesting biochemical questions about the RNA substrate preference of A3B. This is a well-written and timely manuscript which has my support for publication with a few minor suggestions listed below.

RNA editing is not intuitively associated with driving cancer or other diseases. It would be nice to have a hypothesis for this mechanism stated in the introduction, to set the stage for subsequent data and discussion.

We thank the reviewer for the suggestion and have added the following sentence (line 57): “Even though most edits occur in non-coding regions and could have no impact, these modifications may trigger novel splice sites, alter microRNA activity, and/or mRNA stability. Interestingly, it has been shown that the impact of RNA editing on proteome diversity is comparable to, or potentially even greater than, that of somatic mutations (Gassner FJ et al., Peng et al.)”

Line 29-32: RNA editing is a dynamic process, but the RNA edits don't necessarily disappear right after the responsible enzyme is gone. Loss of RNA edits also requires turnover of transcripts. I suggest a clarification of this statement.

We have modified this sentence in the manuscript, (line 85): Unlike DNA mutations, RNA editing is a dynamic process which does not leave a permanent footprint and RNA edits disappear right after the responsible enzymes are no longer expressed or after the turnover of the edited transcripts.

Supp Fig 1E - the dots representing A3B levels on the far right are hard to see and colors are not discernable.

We have included a clearer version of this figure.

Fig 3B - please clarify in the legend and methods how relative contribution is defined. Is this the absolute amount of RNA edits in A3B mice? Or is it measured relative to control mice?

We are sorry for the confusion. RNA editing events included in this study were primarily extracted by removing the matched exome and transcriptomic data for each sample. Subsequently, the RNA editing per trinucleotide profiles for all base substitutions were plot as the absolute amount of RNA edits in A3B mice. Relative contribution refers to the contribution that each single base substitution has to the overall base substitution spectrum in A3B mice. We have clarified this in the figure legends and the methods.

Supp Fig 4 - the color coding is a little confusing because it encompasses both location and type of mutation but makes them mutually exclusive. These categories could co-occur (for example, a missense mutation in the 3'UTR) but the table does not allow for that information to be conveyed.

The reviewer is right. We intended to represent the annotation to better see the distribution of mutations. We have clarified this in the figure and correspondent figure legend, and changed “location” to “annotation”. Please note that Figure S4 is now Figure S5.

**Reviewer #2**: The manuscript by Alonso de la Vega and Colleagues reports the effects of APOBEC3B artificial induction in transgenic mice. The mice were generated by introducing an expression cassette bearing the coding sequence for an APOBEC3B-turboGFP chimera into the ColA1 locus under the control of a TetOn inducible promoter. The experiments were carried out after induction of the APOBEC3B chimera in 4-weeks old mice through doxycycline feeding.

The expression levels of the chimera were compared to the physiological/pathological ones in human tissues to make sure these were in the same order of magnitude. The two main findings are:

- prolonged expression of the APOBEC3B is lethal in mice

- with very high levels of APOBEC3B, its ability to target RNA becomes evident.

Overall, the manuscript is quite interesting: as it asks questions that have been lingering around for a while - specifically, how comparable are APOBEC3A and APOBEC3B, as they share a virtually identical C-terminal domain?  While a more exhaustive answer would have been preferable, the short answer is probably good enough: the two proteins are probably more similar than we would like to acknowledge, with the higher genotoxicity of APOBEC3B being the reason for these differences (APOBEC3B inability to trigger tumors in murine models and the failure to detect APOBEC3B dependent RNA editing in human cells).

Major comments

- As it might be relevant for DNA accessibility, it would be useful to show that localization of the APOBEC3B-turboGFP chimera is the same as that of wild-type APOBEC3B (and how it compares to APOBEC3A).

We thank the reviewer for this comment. We tried to generate cells that express wild-type APOBEC3B without turboGFP. Regrettably, when the plasmid was expressed in bacteria, A3B expression caused mutations in the bacterial DNA that prevented the bacteria to grow, making the amplification of the plasmid unachievable. Other reports have shown that A3B is expressed in the nucleus similar to what we also show in Figure S1C.

- How similar is the phenotype of APOBEC3B expressing mice to those from other genotoxic agents? If specific tissues/pathways that are responsible for the phenotype were identified for other genotoxic agents, it might be worth assessing them also in these mice.

This is an interesting idea. We suppose that, at some threshold, all genotoxic agents will become lethal to mice. We have not been able to address this idea here but parallel studies with animals constitutively expressing APOBEC3B (*Rosa26::CAG-A3Bi*) have demonstrated that this enzyme is capable of driving tumor development (Durfee *et al*., *bioRxiv* doi: <https://doi.org/10.1101/2022.04.26.489523>). Accelerated tumor development requires the enzyme’s catalytic glutamate (E255) and, interestingly, in preliminary studies that are ongoing, APOBEC3B and chemical mutagens appear to have synergistic effects. This work is being pursued by the Harris group at UT Health San Antonio and is expected to take at least another year to complete, and it is therefore well beyond the scope of the present study.

- Considering that RNA-seq experiments were performed using stranded libraries, there is no reason to group together C>U/G>A and A>G/U>C changes. This is quite important, as G>A and U>C changes can be used as internal controls for RNA editing. A useful way to depict the specificity of the RNA editing could be to plot each position with a change through its (average) frequency and its recurrence: only C>U and A>G changes should present a bias.

We thank the reviewer for pointing this out. We created a new R package called plotRNAedits to read mutation files and plot the full 192 context RNA edit profiles. The relevant figures have been included in Figures S3A and S3B for liver and pancreas samples, respectively. Plot RNAedits package is released through github and included in the methods section.

Minor points
- Is there any correlation between RNA editing levels and the expression levels of the edited transcripts – especially in the case of the recurrent sites?

We thank the reviewer for this comment. We have analysed the expression levels (TPMs) of the edited transcripts (for all the transcripts or only the recurrent ones) in liver, as well as in pancreas, and included these data in the revised Figure 4C. In the pancreas all the recurrent edited transcripts are more expressed than the ones that are not edited. Furthermore, all these editing events occur at the 3’UTR, which suggests that the editing might be affecting the stability of the edited transcript.

- It would be useful to add the editing frequency in Table 2 as well

We have now added the frequency in Table 2.

- It would be useful if the Authors could discuss how they explain the specificity/frequency of the editing at some of the sites with the requirement for a very high APOBEC3B expression. I would imagine that, with such specificity/frequency, even lower APOBEC3B expression would be sufficient to detect the editing.

This is another very interesting point made by the reviewer. In fact, editing does not occur when the expression of A3B is low. Apart from the one reported in this manuscript, we have generated an additional mouse model that expresses the rtTA transactivator under the control of the Rosa26 promoter: *TetO-A3B-tGFP/Rosa26-rtTA* (reported in Durfee *et al*., *bioRxiv* doi: <https://doi.org/10.1101/2022.04.26.489523>). After induction of doxycycline for 7 days, these mice expressed low levels of A3B in most of the tissues, in line with previous reports suggesting that the Rosa26 promoter has variable activity in different mouse tissues. Recurrent liver RNA editing events were validated in the livers of 2 mice after 7 days on doxycycline, in which no C-to-U changes were detected in any of the 7 positions examined, indicating that RNA editing activity requires high A3B expression levels. One hypothesis for this could be that A3B has more affinity for DNA substrates than RNA. Previous studies have demonstrated that A3A and APOBEC1 have a preferential targeting of DNA over RNA (<https://doi.org/10.1073/pnas.1306345110>, <https://doi.org/10.1093/nar/gkt945>, <https://doi.org/10.1021/acschembio.1c00919>). Therefore, low A3B levels could potentially focus on specific DNA substrates, whereas elevated A3B levels could deaminate a greater number of targets, including RNA molecules.

- page 6. "Feed ab libitum" should be "fed ad libitum"

We apologise for the mistake which we have now corrected and thank the reviewer for pointing this out.

**Second round of review**

**Reviewer 1**

Alonso de la Vega and colleagues have addressed all points made by reviewers adequately. Overall, the manuscript is appropriate for publication and is likely to be well cited given its novelty (there are other similar stories of A3B as an RNA editor in preparation now, so it is exciting to read the first paper to define this concept). In my final review of this manuscript, I realized that there are no data showing RNA editing (by RNA seq or Sanger validation) from control animals. In particular, Fig 3B and S3A-B should take into account RNA editing in control animals. In my opinion, the best option is to subtract control RNA edits from the RNA editing signatures in A3B tissues similar to how DNA editing was handled (see workflow in Fig 3A). It would also be reasonable to generate RNA editing signatures from controls and show them next to the A3B mice signatures. Line 171 mentions that “higher levels of C>U edits were only evident in A3B-expressing tissues and not in controls” but it is unclear what controls means here: control mice? Control tissues in A3B-tGFP mice with low/no A3B expression? I think the data regarding A3B as the etiology of RNA editing are convincing based on the pulse/off studies and A3B-E255Q studies which show no editing. But in the interest of transparency and data availability, it is important to include the RNA seq from control animals in this manuscript. From what I can tell, two control mice underwent RNA seq of liver and pancreas so I hope inclusion of these data isn’t too much trouble.

**Authors’ response to reviewers**

1. We have pointed out in the manuscript a control mouse (no A3B expression) that was used for Sanger sequence validation. “*We observed that 4 days of doxycycline administration were sufficient to trigger A3B expression (Additional file 1: Fig. S7A) and consequently induce A3B-driven RNA editing events in 6 out of the 7 positions examined in A3B samples but not in control (Fig. 6B and Additional file 3: Table S2)”.* We also modified the corresponding figure legend: **Fig.6B)**
2. We have changed the sentence: “*higher levels of C>U edits were only evident in A3B-expressing tissues and not in controls*”” that now reads: “*high levels of C-to-U RNA editing were present in A3B-expressing tissues compared to controls*”.
